# Supplementary material for: Single cell dual-omic atlas of the human developing retina
Source: Nat Commun. 2024 Aug 9;15:6792. doi: 10.1038/s41467-024-50853-5 (PMC11310509; doi:10.1038/s41467-024-50853-5)
Supplement: Supplementary file 1 — Supplementary Information [file 41467_2024_50853_MOESM1_ESM.pdf]

Supplementary Information: Single Cell Dual-omic Atlas of the Human Developing Retina

Zhen Zuo[1,2,+], Xuesen Cheng[1,+], Salma Ferdous[1,+], Jianming Shao[1], Jin Li[1], Yourong Bao[1], Jean Li[1], Jiaxiong Lu[1], Antonio Jacobo Lopez[3], Juliette Wohlschlegel[4], Aric Prieve[4], Mervyn G Thomas[5], Thomas A Reh[4], Yumei Li[1], Ala Moshiri[3], and Rui Chen[1,2,6,*]

[1]HGSC, Department of Molecular and Human Genetics, Baylor College of Medicine, 1 Baylor Plaza, Houston, 77030, Texas, United States.
[2]Graduate Program in Quantitative and Computational Biosciences, Baylor College of Medicine, 1 Baylor Plaza, Houston, 77030, Texas, United States.
[3]Department of Ophthalmology & Vision Science, UC Davis School of Medicine, 4860 Y St, Sacramento, 95817, California, United States.
[4]Department of Biological Structure, University of Washington, 1410 NE Campus Pkwy, Seattle, 98195, Washington, United States.
[5]Ulverscroft Eye Unit, School of Psychology and Vision Sciences, The University of Leicester, Leicester, LE1 7RH, United Kingdom.
[6]Verna and Marrs McLean Department of Biochemistry and Molecular Biology, Baylor College of Medicine, 1 Baylor Plaza, Houston, 77030, Texas, United States.
[+]these authors contributed equally to this work
[*]Corresponding Author: Rui Chen (ruichen@bcm.edu)

Supplementary Figures and legends

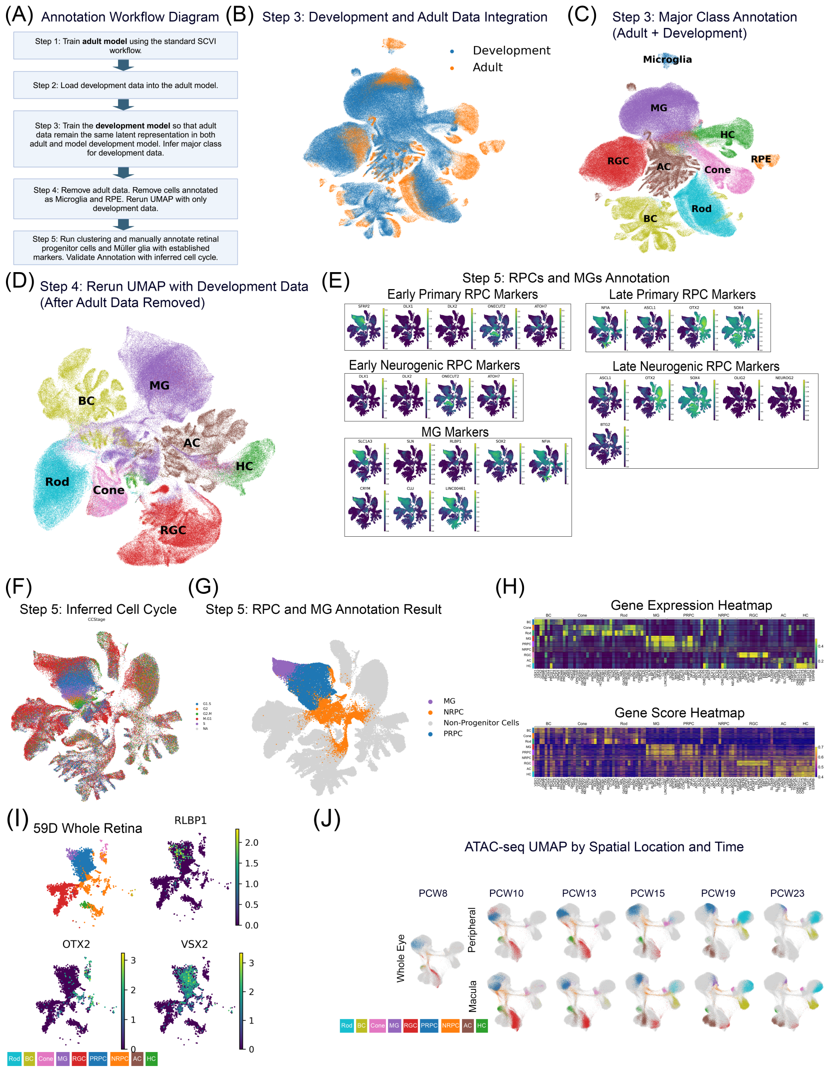

Supplementary Figure 1: Major Class Annotation

(A) Major class annotation workflow diagram. Step 1-3 are reference mapping with scvi-tools. In step 4 and 5, adult data was removed, and development data was clustered to annotate PRPC, NRPC, and MG based on established markers. (B) UMAP of integrated development and adult data from step 3. UMAP was colored by sample source, which is either from adult samples in orange, or development sample in blue. (C) UMAP of inferred major class annotation for development data from step 3. At this step, all progenitors

were labeled as MG, and these annotations will be refined using established RPC/MG markers in step 4 and step 5. (D) UMAP of development data only from step 4. At this step, cells from adult samples have been removed. In addition, cells labeled as "Microglia" or "RPE" were removed. All progenitors were labeled as MG, and these annotations will be refined using established RPC/MG markers in subsequent steps. (E) UMAPs showing the expression of established markers for all cells provisionally labeled as MG. Then, PRPC, NRPC, and MG are manually annotated based on clustering and expression patterns of these established markers (see Methods) through clustering. (F) UMAP of inferred cell cycle. Cells that cannot be assigned to any cell cycle were labeled in gray (as "NA"). (G) UMAP of annotation results of PRPCs, NRPCs, and MGs after Step 5. Non-progenitor cells were labeled in gray. (H) Panel 1: Established marker gene (see Methods) expression heatmap of all cells, where each row represents the gene expression profile of an individual cell. The cells were randomly down sampled to ensure an equal number of cells for each major class, and then the whole major class were grouped and ordered by similarities in gene expression; Panel 2: ATAC-seq gene score heatmap of all cells, using the same marker genes as in the panel 1. Gene scores were normalized and imputed for better visualization. (I) UMAP for 59 days post conception, whole retina sample. UMAP of MG and BC maker genes. (J) ATAC-seq UMAP separated by spatial location and clock time and colored by major class. (A) was created with `BioRender.com`.

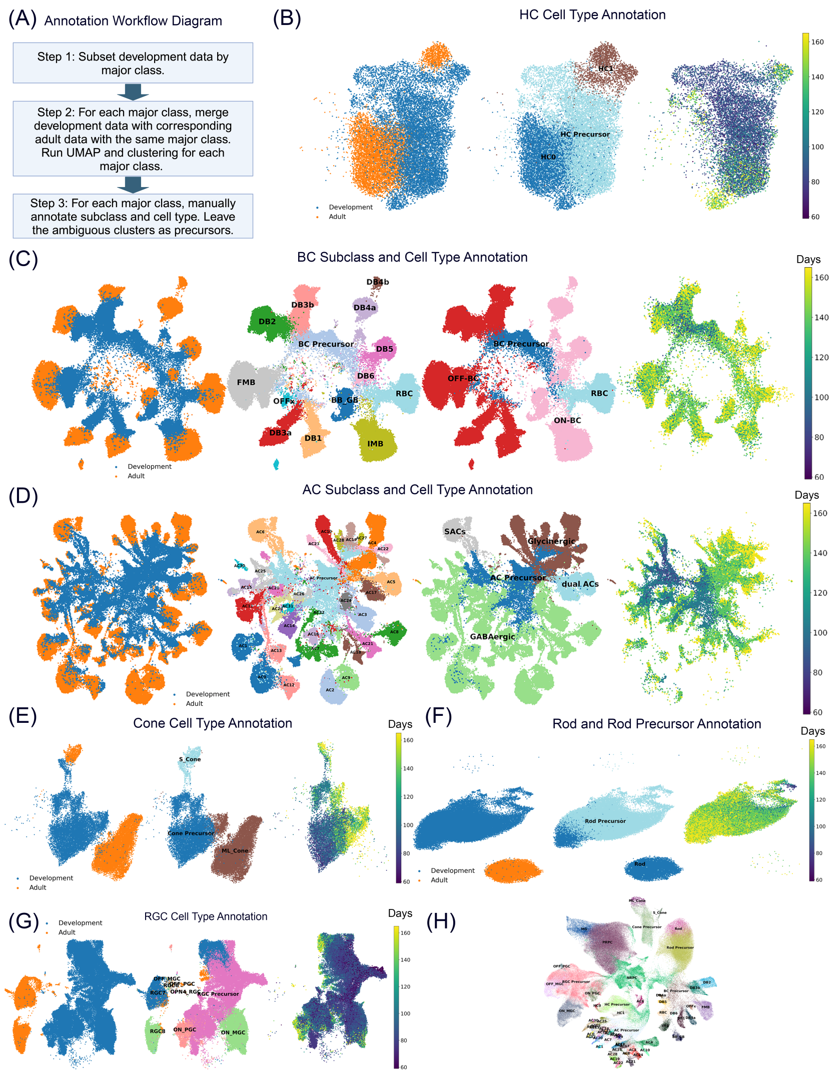

Supplementary Figure 2: Subclass and Cell Type Annotation

(A) Subclass and cell type annotation workflow diagram. First, adult cells and development cells from the same corresponding major class were integrated together. Second, developmental cells were annotated manually based on gene expression similarities on UMAPs. (B)-(G) Subclass annotation process for HCs, BCs, ACs, cones, rods, and RGCs. Firstly, cells labeled as each specific major class in the developmental data were separated and then integrated with their corresponding adult cells. Subsequently, subclass annotations were manually added based on clustering. Any cells that could not be confidently assigned

to any specific subclass were labeled as precursors. UMAPs are color-coded according to their sample source (either adult or developmental), annotated subclass (if subclass exist), annotated cell type, and respective post-conception days. (H) UMAP of annotated cell types for all development data. (A) was created with `BioRender.com`.

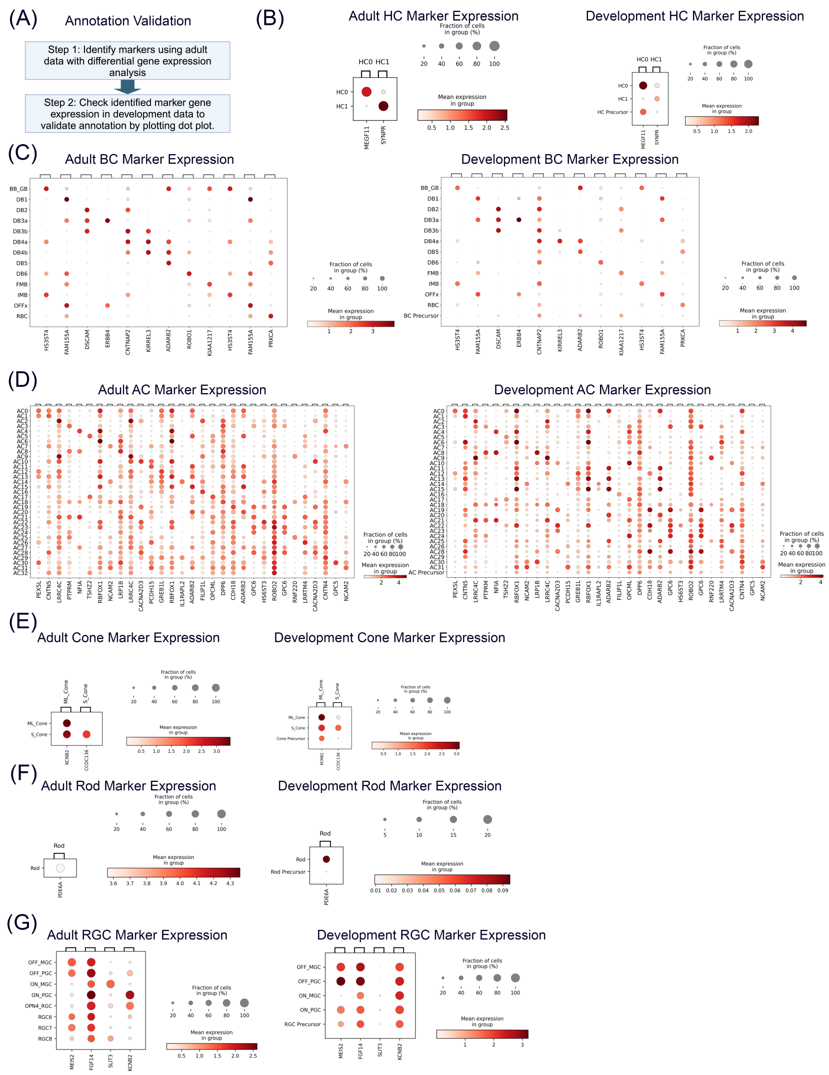

Supplementary Figure 3: Subclass Annotation Validation with Marker Gene Expression

(A) Cell type annotation validation workflow diagram: First, marker genes were identified using adult data through a differential gene expression analysis for each major class. Subsequently, for each major class, developmental precursors and annotated data were visualized with those markers to illustrate gene expression patterns. Ideally, annotated development data should have similar gene expression patterns of those markers as adult data. The analysis was then tested using the method="t-test_overestim_var", which stands for overestimating the two-side variance in each group t-test. P-values were corrected using the

Benjamini-Hochberg method, specifying corr_method="benjamini-hochberg". (B-G) Dot plots showing gene expression patterns in adult and developmental data for HCs, BCs, ACs, cones, rods, and RGCs. (A) was created with `BioRender.com`.

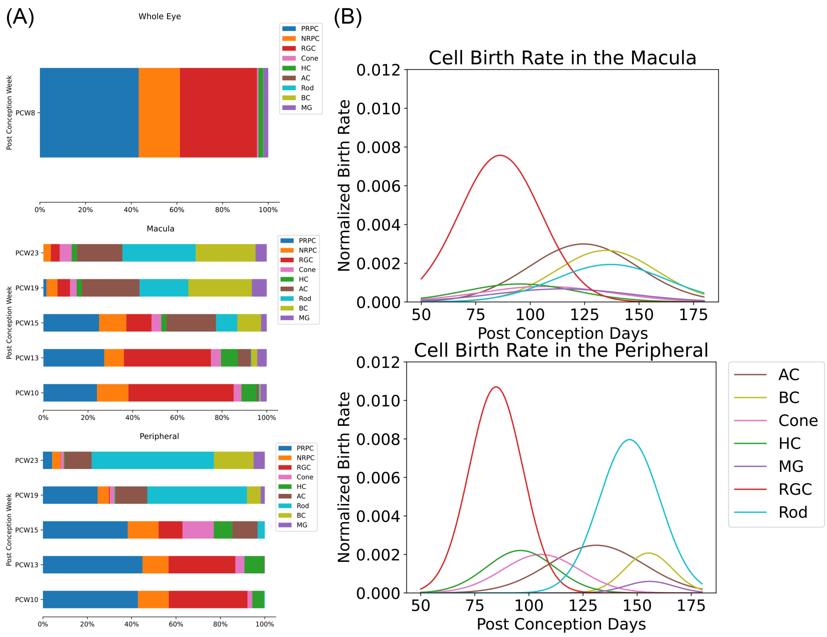

Supplementary Figure 4: Cell Proportions

(A) Proportions of major classes grouped by PCW for whole eye, macula, and periphery. (B) Estimated density plot of major classes. The x-axis represents post-conception days, and the y-axis is the normalized birth rate estimated for that time point. The area under the curve for each plot indicates the overall proportion of each major class.

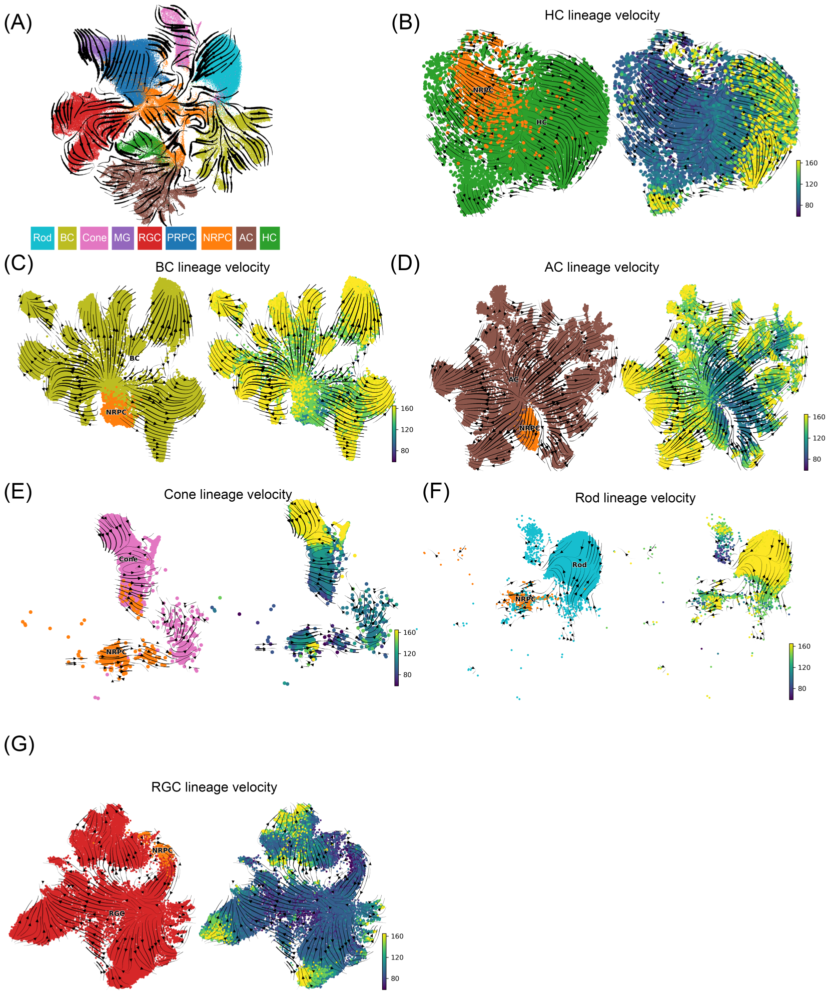

Supplementary Figure 5: Major Class Velocities

(A) Stream plot showing velocities (estimated from RNA-seq) for all cells. To estimate velocities for all cells, only RNA-seq data was utilized to streamline computations and accommodate computing resource limitations. (B)-(G) Stream plot showing velocities (estimated from RNA-seq and ATAC-seq) for HCs, BCs, ACs, cones, rods, and RGCs. UMAPs were colored by annotated major class and days post conception.

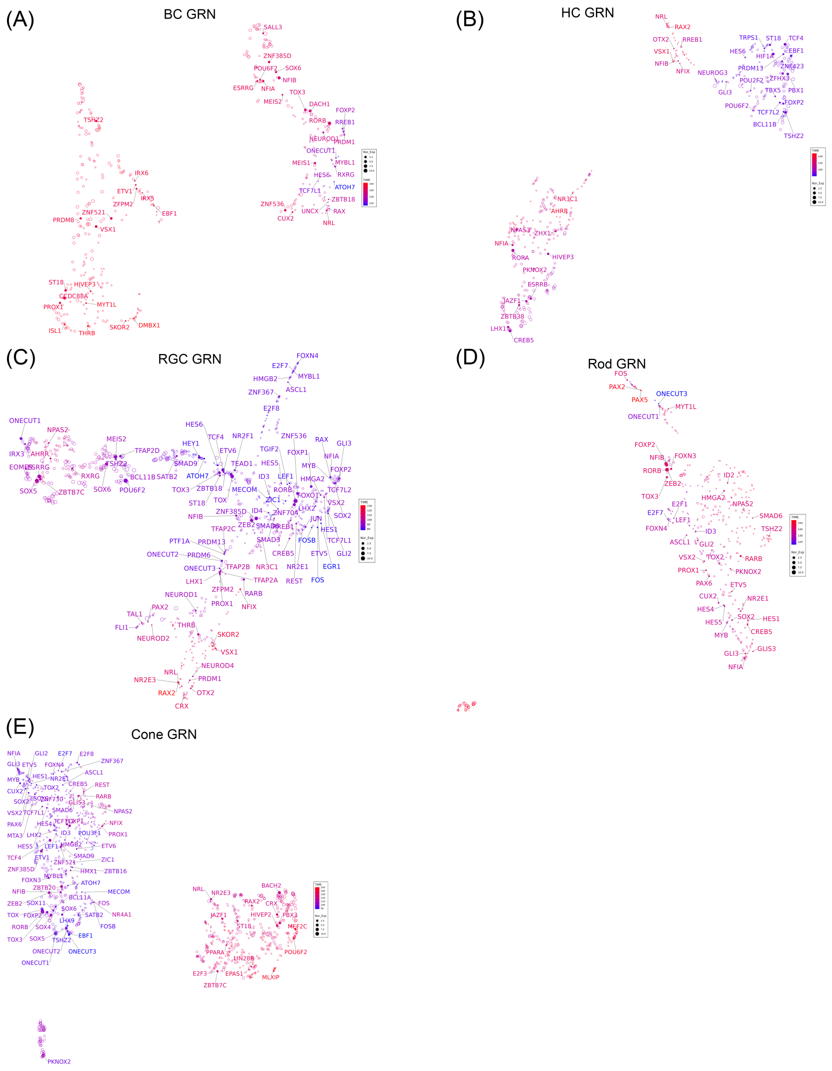

Supplementary Figure 6: Gene Regulatory Network

(A) –(E) UMAP embedding of the inferred TF network based on weighted co-expression and inferred interaction strength between TFs. The UMAP is different from the gene expression UMAP. To infer the GRN, all cells from the corresponding lineage were used. For example, in the BC lineage, all BCs, BC precursors, and BC progenitors were used for GRN inference. Color and size represent the expression-weighted days post-conception and normalized gene expression levels of each TF, respectively.

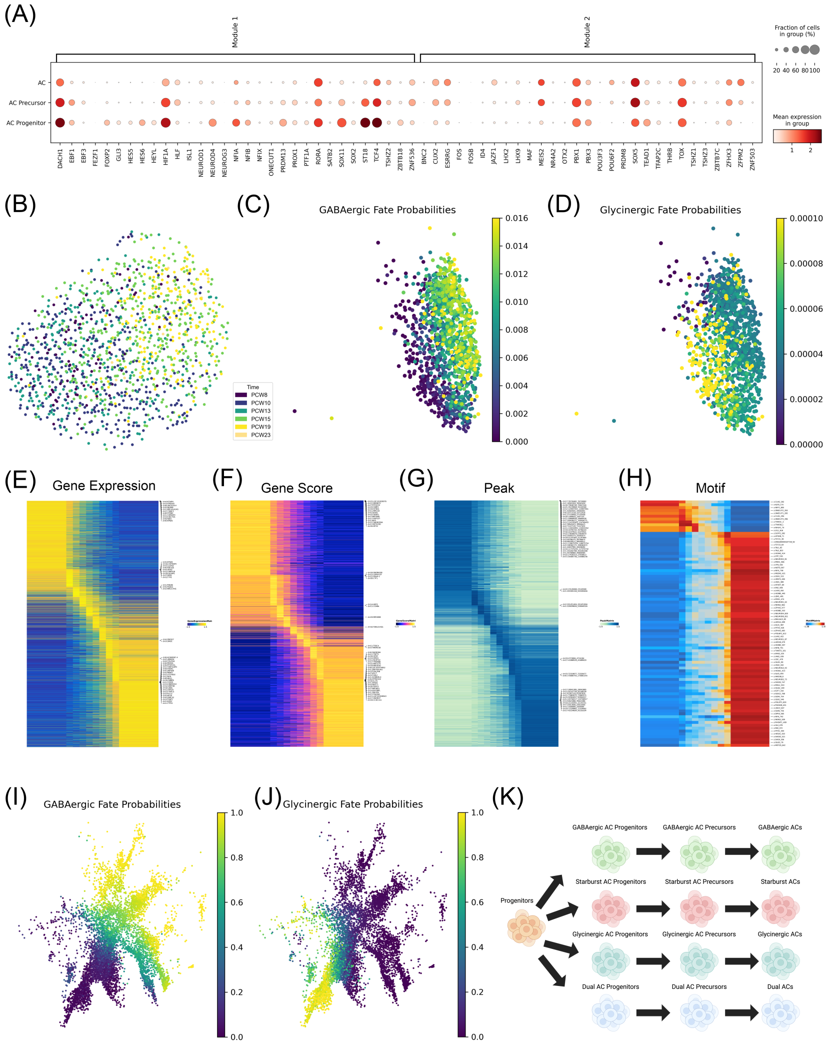

Supplementary Figure 7: AC Branch Gene Expression and Chromatin Accessibility Dynamics

(A) Dot plot of TFs identified in AC branch GRNs. TFs were clustered by k-mean clusters and showing expression in ACs, AC precursors, and AC progenitors. The analysis was then tested using the method="t-test_overestim_var", which stands for overestimating the two-side variance in each group t-test. P-values were corrected using the Benjamini-Hochberg method, specifying corr_method="benjamini-hochberg". (B) ATAC-seq UMAP of AC progenitors. Cells were colored by PCWs. This UMAP is not from the global ATAC-seq UMAP for all cells. The UMAP was run only on AC progenitors. (C) Estimated fate probability for

GABAergic fate of AC progenitors. (D) Estimated fate probability for Glycinergic fate of AC progenitors. (E) Gene expression heatmap of AC progenitors from early to late stage. Cells were ordered by PCW groups. (F) Gene score heatmap of AC progenitors from early to late stage. Cells were ordered by PCW groups. (G) Peak accessible heatmap of AC progenitors from early to late stage. Cells were ordered by PCW groups. (H) Motif deviation of AC progenitors from early to late stage. Cells were ordered by PCW groups. For (E-G), cells were arranged according to the pseudotime inferred from the ATAC-seq trajectory. Features were selected based on the Pearson correlation between gene expression and motif deviation, with a threshold set greater than 0.3. (I) Estimated fate probability for GABAergic fate of AC precursors. (J) Estimated fate probability for Glycinergic fate of AC precursors. (K) Hierarchical model for AC fate commitment during development of the human retina. (K) was created with `BioRender.com`.

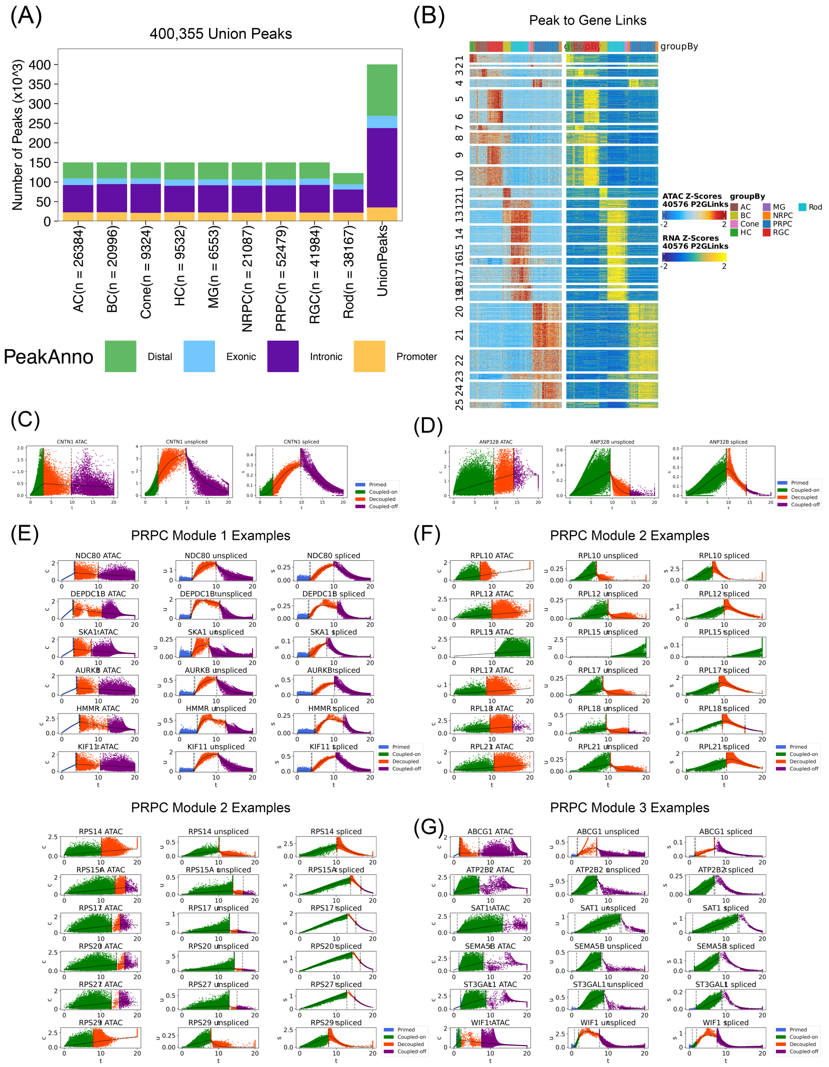

Supplementary Figure 8: Chromatin Accessibility and Gene Expression Patterns

(A) Number of peaks identified in all cells. Peaks were identified in each major class separately and then union together to get union peak sets. (B) Peak-to-Gene linkage identified in all cells. Gene-peak pairs were clustered based on gene expression and pair accessibility among all cells. (C) Example of a gene that shows all three cell states across PRPCs. The three states are coupled-on, decoupled, and coupled-off. For the decoupled stage, ATAC level is going down, while unspliced and spliced levels are going up. (D) Another example of a gene that shows all three cell states across PRPCs. The three states are coupled-on,

decoupled, and coupled-off. For the decoupled stage, ATAC level is going up, while unspliced and spliced levels are going down. (E) Plots for PRPC Module 1 Genes. Observed values for c (left), u (middle) and s (right) plotted as a function of latent time and colored by state assignment. Vertical lines indicate inferred switch times. (F) Plots for PRPC Module 2 genes. Observed values for c (left), u (middle) and s (right) plotted as a function of latent time and colored by state assignment. Vertical lines indicate inferred switch times. (G) Plots for PRPC Module 3 Genes. Observed values for c (left), u (middle) and s (right) plotted as a function of latent time and colored by state assignment. Vertical lines indicate inferred switch times.

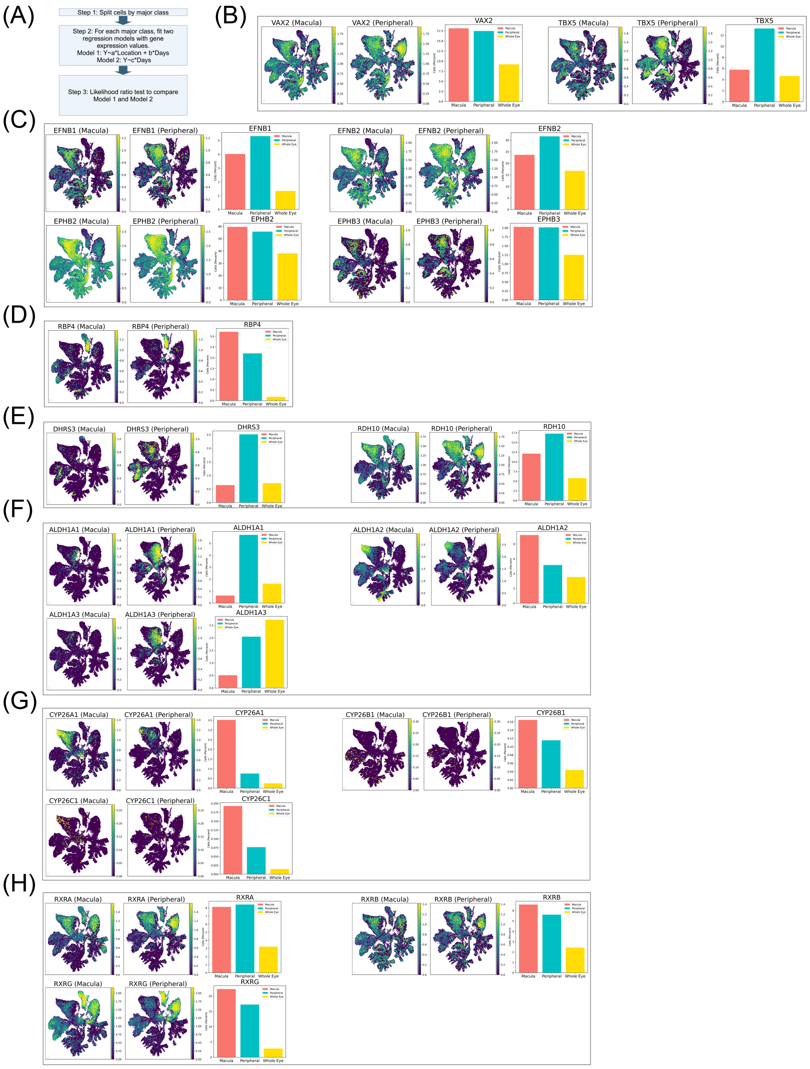

Supplementary Figure 9: Identification of Differentially Expressed Genes Between Macula and Periphery

(A) Differently expressed gene identification workflow diagram. For each major class, regression models were used for regression out the effects of "Days" during DEGs test between macula and peripheral. Then, a likelihood ratio test was performed for each group of cells by comparing the effects of "Region" and regressing out the effects of "Days", which is a vector of integer representing sample age (days post conception). (B) Gene expression UMAPs and bar charts for number of cells have non-zero gene expression of VAX2 and TBX5 in macula, periphery, and whole eye. (C) Gene expression UMAPs and bar charts for number

of cells have non-zero gene expression of genes coding ephrin protein (*EFNB1*, *EFNB2*) and ephrin receptors (*EPHB2*, *EPHB3*) in macula, periphery, and whole eye. (D) Gene expression UMAPs and bar charts for number of cells have non-zero gene expression of Retinol binding protein 4 (RBP4) in macula and periphery, and whole eye. (E) Gene expression UMAPs and bar charts for number of cells have non-zero gene expression of dehydrogenase/reductase-SDR family member 3 (*DHRS3*) and retinol dehydrogenase-10 (*RDH10*) in macula and periphery, and whole eye. (F) Gene expression UMAPs and bar charts for number of cells have non-zero gene expression of aldehyde dehydrogenase-1A (*ALDH1A*) enzymes in macula and peripheral, and whole eye. (G) Gene expression UMAPs and bar charts for number of cells have non-zero gene expression of CYP26 enzymes in macula and periphery, and whole eye. (H) Gene expression UMAPs and bar charts for number of cells have non-zero gene expression of RA receptors in macula and periphery, and whole eye. (A) was created with `BioRender.com`.

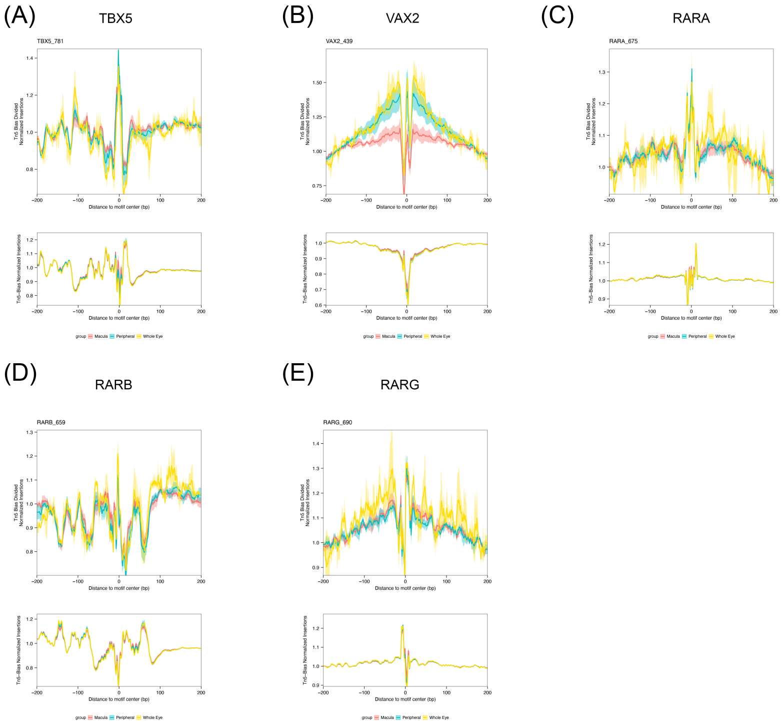

Supplementary Figure 10: Compare Motif Binding for TFs Between Macula and Periphery

(A)-(E) Footprinting to estimate TFs binding and compare signal of Tn5 insertion events in macula and periphery regions. The red line is the signal intensity in the macula region and the blue line is the signal intensity in the peripheral. For each pot, the x range is 250 base pair around the corresponding motif center. TFs are *TBX5*, *VAX2*, *RARA*, *RARB*, and *RARG*.

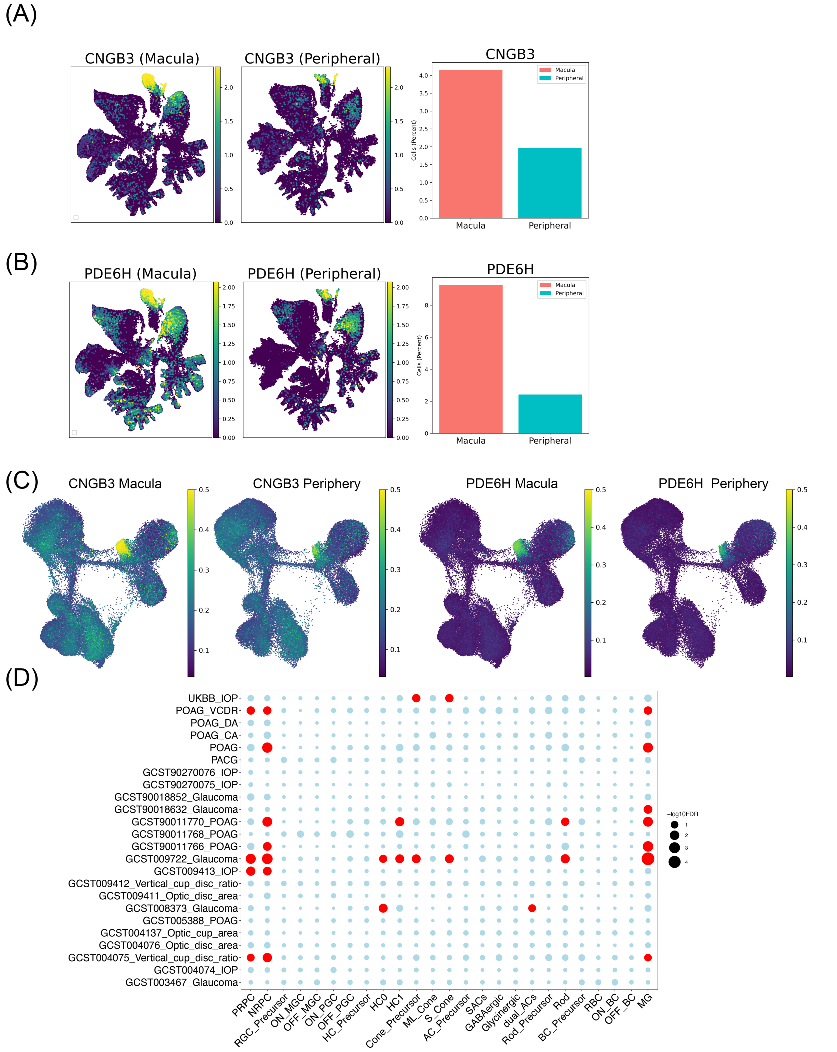

Supplementary Figure 11: Macular Hypoplasia Related Gene Expression

(A) Gene expression UMAPs and bar charts for number of cells have non-zero gene expression of *CNGB3*. (B) Gene expression UMAPs and bar charts for number of cells have non-zero gene expression of *PDE6H*. (C) ATAC-seq UMAP colored by gene score of *CNGB3* and *PDE6H* in the macula and periphery. The UMAPs are the same as Fig. 1C. (D) The subclass enrichment of 24 eye-related GWAS traits based on gene expression from snRNA-seq data. IOP (intraocular pressure). PACG (primary angle closure glaucoma). POAG (primary open-angle glaucoma); CA (cup area of optic nerve); DA (disc area of optic nerve);

VCDR (vertical cup-disc ratio of optic nerve). To test GWAS traits enrichment, two-side F-test was applied to compute the p-values. The Benjamini-Hochberg procedure was applied. Significant terms are highlighted in red (FDR <0.05).
