# Supplementary material for: Single cell dual-omic atlas of the human developing retina
Source: Nat Commun. 2024 Aug 9;15:6792. doi: 10.1038/s41467-024-50853-5 (PMC11310509; doi:10.1038/s41467-024-50853-5)
Supplement: Supplementary file 3 — Description of additional supplementary files [file 41467_2024_50853_MOESM3_ESM.pdf]

Description of Additional Supplementary Files

Supplementary Data 1

Summary of all sample information and statistics from the CellRanger report. Donor ID is a factor variable indicating which donor the sample was collected from.

Supplementary Data 2

Summary of the QC process implemented in this study. Each single nucleus underwent four filtering steps to prepare them for downstream analysis.

Supplementary Data 3

The top marker genes identified for each major class. Within each major class, the top 2,000 highly variable genes were first calculated, and differential expression analysis was performed on those highly variable genes to obtain summary statistics. Two-side overestimates variance t-test was applied to 226,506 cells to compare gene expression values among all major classes. The Benjamini-Hochberg procedure was applied to decrease the false discovery rate.

Supplementary Data 4

Summary of the number of cells identified for each cell type. The unidentified cell types were summarized in the second tab.

Supplementary Data 5

Gene Module Analysis results on PRPCs. The first tab summarizes the gene module assignment results. In total, genes were assigned to three distinct modules. Genes that could not be assigned to any modules were labeled as -1. The second, third, and fourth tabs contain gene ontology analysis results for genes identified in each module. For the gene ontology analysis, the one-side hypergeometric test was used to measure the significance of a functional term in the input gene list. Adjusted p-values were calculated from Benjamini-Hochberg procedure.

Supplementary Data 6

Gene regulatory network inference result. The table summarizes TF, target gene, regulation region, estimated effects, and summary statistics of the generalized linear model fitting. The coefficients obtained from the fitting process underwent statistical significance testing via analysis of variance (ANOVA). In total, 226,506 cells were used to compute the GRNs. Benjamini–Hochberg method was

applied to account for multiple comparisons. A significance threshold of 0.05 was then utilized to determine statistical significance.

Supplementary Data 7

Top marker genes identified in NRPCs. NRPCs were grouped by inferred fate. Gene ontology analysis was then performed on the identified marker genes. To identify differentially expressed genes, the two-side overestimates variance t-test was applied to different fate groups among 21,087 cells. For the gene ontology analysis, the one-side hypergeometric test was used to measure the significance of a functional term in the input gene list (100 genes for each annotated fate group). Adjusted p-values were calculated from Benjamini-Hochberg procedure.

Supplementary Data 8

Functional prediction results for TFs identified in NRPCs. The second tab summarizes previously published loss-of-function effects on those TFs, serving as ground truth.

Supplementary Data 9

Gene expression matrix, gene score matrix, peak matrix, and motif matrix identified in AC progenitors. Each row represents a feature, and each column represents values at different time points, ranging from early to late. Only features that are dynamic with time were shown here. Dynamic features were selected based on the Pearson correlation between gene expression and motif deviation, with a threshold set greater than 0.3.

Supplementary Data 10

Tab 1 shows all peaks identified in our data. Tab 2-10 show differential accessed regions identified for each one of the major classes. Tab 11 shows peaks with peak-to-gene links. Tab 12 and tab 13 showed regions with adult histone modification signals. Marker peaks were identified using the one-side Wilcoxon rank-sum test using the Benjamini-Hochberg correction, applying a threshold of false discovery rate \textless 0.01 and a Log2 Fold Change \textgreater 1.

Supplementary Data 11

Summary of differentially expressed genes identified in each major class between the macula and periphery. Two models were used to identify these genes. One model is the location model, which tests if gene expression is correlated with location as a covariate. The other model is the likelihood model, where the full model includes both location and days as covariates, and the reduced model includes only days as input variables for linear regression model fitting. The two-side Wald test was used to calculate p values, and false discovery rates were estimated with Benjamini-Hochberg procedure.

Supplementary Data 12

Gene ontology analysis results of the DEGs identified in PRPCs between the macula and periphery. The one-side hypergeometric test was used to measure the significance of a functional term in the input gene list. Adjusted p-values were calculated from Benjamini-Hochberg procedure.
