# Supplementary material for: Single cell dual-omic atlas of the human developing retina
Source: Nat Commun. 2024 Aug 9;15:6792. doi: 10.1038/s41467-024-50853-5 (PMC11310509; doi:10.1038/s41467-024-50853-5)
Supplement: Supplementary file 16 — Reporting Summary [file 41467_2024_50853_MOESM16_ESM.pdf]

nature portfolio

Corresponding author(s): Rui Chen

Last updated by author(s): Jul 20, 2024

Reporting Summary

Nature Portfolio wishes to improve the reproducibility of the work that we publish. This form provides structure for consistency and transparency in reporting. For further information on Nature Portfolio policies, see our Editorial Policies and the Editorial Policy Checklist.

Statistics

For all statistical analyses, confirm that the following items are present in the figure legend, table legend, main text, or Methods section.

n/a	Confirmed	
☐	☒	The exact sample size (*n*) for each experimental group/condition, given as a discrete number and unit of measurement
☒	☐	A statement on whether measurements were taken from distinct samples or whether the same sample was measured repeatedly
☐	☒	The statistical test(s) used AND whether they are one- or two-sided<br>*Only common tests should be described solely by name; describe more complex techniques in the Methods section.*
☒	☐	A description of all covariates tested
☐	☒	A description of any assumptions or corrections, such as tests of normality and adjustment for multiple comparisons
☐	☒	A full description of the statistical parameters including central tendency (e.g. means) or other basic estimates (e.g. regression coefficient) AND variation (e.g. standard deviation) or associated estimates of uncertainty (e.g. confidence intervals)
☐	☒	For null hypothesis testing, the test statistic (e.g. *F*, *t*, *r*) with confidence intervals, effect sizes, degrees of freedom and *P* value noted<br>*Give P values as exact values whenever suitable.*
☒	☐	For Bayesian analysis, information on the choice of priors and Markov chain Monte Carlo settings
☒	☐	For hierarchical and complex designs, identification of the appropriate level for tests and full reporting of outcomes
☒	☐	Estimates of effect sizes (e.g. Cohen's *d*, Pearson's *r*), indicating how they were calculated

Our web collection on statistics for biologists contains articles on many of the points above.

Software and code

Policy information about availability of computer code

Data collection	Cell Ranger ARC 1.0.0, and Cell Ranger ARC 2.0.0
Data analysis	Seurat 4.4.0, Scanpy 0.1.0, Pando 1.0.3, velocyto 0.17.17, CellRank 1.5.1, MultiVelo 0.1.3, scVelo 0.3.2, scvi-tools 1.1.0, ArchR 1.0.1, Hotspot 1.1.1, g:Profiler, GREAT 4.0.4, and custom algorithms at https://github.com/zhenzuo2/Human-Developmental-Retina-Atlas

For manuscripts utilizing custom algorithms or software that are central to the research but not yet described in published literature, software must be made available to editors and reviewers. We strongly encourage code deposition in a community repository (e.g. GitHub). See the Nature Portfolio guidelines for submitting code & software for further information.

Data

Policy information about availability of data

All manuscripts must include a data availability statement. This statement should provide the following information, where applicable:

- Accession codes, unique identifiers, or web links for publicly available datasets
- A description of any restrictions on data availability
- For clinical datasets or third party data, please ensure that the statement adheres to our policy

The RNA-seq and ATAC-seq raw data generated in this study can be accessed at National Center for Biotechnology Information (NCBI) with Sequence Read Archive (SRA) accession ID SRP510712 and with link https://trace.ncbi.nlm.nih.gov/Traces/study/?acc=SRP510712&o=acc_s%3Aa. A copy of raw data have been deposited

in the HCA Data Portal - Human Cell Atlas, under accession code 581de139-461f-4875-b408-56453a9082c7 and with link https://explore.data.humancellatlas.org/projects/581de139-461f-4875-b408-56453a9082c7.

The processed data are available at CZ CELLxGENE Discover with accession code 5900dda8-2dc3-4770-b604084eac1c2c82 and with link https://cellxgene.cziscience.com/collections/5900dda8-2dc3-4770-b604-084eac1c2c82 . The count matrix for all sequencing data is available at Gene Expression Omnibus, with accession code GSE268630 and with link https://www.ncbi.nlm.nih.gov/geo/query/acc.cgi?acc=GSE265801. The binned ATAC-seq peak signaling data used in this study are available in the UCSC Genome Browser Home under session ID HumanDevelopingRetinaAtlas with link https://genome.ucsc.edu/s/zhenzuo2/HumanDevelopingRetinaAtlas.

Adult data used for cell major class annotation and cell subclass annotation is provided in Zenodo https://doi.org/10.5281/zenodo.10806575. Adult differentially accessible regions, adult histone modification regions, inferred gene regulatory network model, recovered dynamic models, identified differentially expressed genes model, and bigwig files for ATAC are provided in Zenodo with link https://doi.org/10.5281/zenodo.10866348.

Source data are provided with this paper as a Source Data file.

Research involving human participants, their data, or biological material

Policy information about studies with human participants or human data. See also policy information about sex, gender (identity/presentation), and sexual orientation and race, ethnicity and racism.

Reporting on sex and gender

This research complies with tenets of the declaration of Helsinki. The study was reviewed and approved by the UC Davis Institutional Review Board (IRB) (IRB ID: 903054-1). The use of discarded de-identified human fetal retinal tissue was approved by the UC Davis Stem Cell Research Oversight committee (SCRO protocol#1171, initial approval 12/16/2019). Human ocular tissues were obtained from discarded de-identified fetal waste from elective abortions. The patients from whom the tissues were derived were informed and freely agreed for them to be used for research purposes. Only tissues from women agreeing to donate it for scientific research were used in this study. Their agreement for research was documented in the medical chart. Patients were not compensated. Due to the early age of tissue procurement from elective abortions, it was not feasible to identify the sex at the time of tissue collection. Fetal tissues were collected masked to sex, and the sex of each sample was determined subsequently by genetic sequencing. Sex was determined by the relative gene expression abundance of XIST and DDX3Y. XIST is only expressed in females, and DDX3Y is only expressed in males. Due to the scarcity of tissue samples at each age, it was not feasible to achieve large sample sizes of each sex at each fetal stage. Therefore, all available samples were included in the analysis. The data was collected from 7 male and 7 female

Reporting on race, ethnicity, or other socially relevant groupings

Due to the early age of tissue procurement from elective abortions, this information is not Available.

Population characteristics

Due to the early age of tissue procurement from elective abortions, this information is not Available.

Recruitment

Only tissues from women agreeing to donate it for scientific research were used in this study. Their agreement for research was documented in the medical chart.

Ethics oversight

This research complies with tenets of the declaration of Helsinki. The study was reviewed and approved by the UC Davis Institutional Review Board (IRB) (IRB ID: 903054-1). The use of discarded de-identified human fetal retinal tissue was approved by the UC Davis Stem Cell Research Oversight committee (SCRO protocol#1171, initial approval 12/16/2019).

Note that full information on the approval of the study protocol must also be provided in the manuscript.

Field-specific reporting

Please select the one below that is the best fit for your research. If you are not sure, read the appropriate sections before making your selection.

☒ Life sciences ☐ Behavioural & social sciences ☐ Ecological, evolutionary & environmental sciences

For a reference copy of the document with all sections, see nature.com/documents/nr-reporting-summary-flat.pdf

Life sciences study design

All studies must disclose on these points even when the disclosure is negative.

Sample size

28 samples from 14 donors. For each donor, macular and peripheral retina was collected from the whole retina. No sample size calculation was performed. The sample sizes were chosen based on previous literature with similar experimental paradigms(DOI: 10.1016/j.devcel.2020.04.009).

Data exclusions

No data exclusions.

Replication

For each donor, two samples were obtained for replications. All attempts to repeat the experiment were successful.

Randomization

This is not relevant to our study because samples were grouped based on the spatial location where the tissue was located. Both locations for all samples were collected, which cannot be randomized.

Blinding

During the data collection process, the people who performed the experiment were not aware of the sample group information. For each sample, the sample name was a random number or letters without any meaning.

Reporting for specific materials, systems and methods

We require information from authors about some types of materials, experimental systems and methods used in many studies. Here, indicate whether each material, system or method listed is relevant to your study. If you are not sure if a list item applies to your research, read the appropriate section before selecting a response.

Materials & experimental systems

n/a	Involved in the study
☒	☐ Antibodies
☒	☐ Eukaryotic cell lines
☒	☐ Palaeontology and archaeology
☒	☐ Animals and other organisms
☒	☐ Clinical data
☒	☐ Dual use research of concern
☒	☐ Plants

Methods

n/a	Involved in the study
☒	☐ ChIP-seq
☒	☐ Flow cytometry
☒	☐ MRI-based neuroimaging

Plants

Seed stocks

Report on the source of all seed stocks or other plant material used. If applicable, state the seed stock centre and catalogue number. If plant specimens were collected from the field, describe the collection location, date and sampling procedures.

Novel plant genotypes

Describe the methods by which all novel plant genotypes were produced. This includes those generated by transgenic approaches, gene editing, chemical/radiation-based mutagenesis and hybridization. For transgenic lines, describe the transformation method, the number of independent lines analyzed and the generation upon which experiments were performed. For gene-edited lines, describe the editor used, the endogenous sequence targeted for editing, the targeting guide RNA sequence (if applicable) and how the editor was applied.

Authentication

Describe any authentication procedures for each seed stock used or novel genotype generated. Describe any experiments used to assess the effect of a mutation and, where applicable, how potential secondary effects (e.g. second site T-DNA insertions, mosiacism, off-target gene editing) were examined.
